# Supplementary material for: From sequence to enzyme mechanism using multi-label machine learning
Source: BMC Bioinformatics. 2014 May 19;15:150. doi: 10.1186/1471-2105-15-150 (PMC4229970; doi:10.1186/1471-2105-15-150)
Supplement: Additional file 2 — Java code of ml2db. Additional file ml2db_code.tar.gz contains the Java source code to run the multi-label machine learning experiments and save the results to database. The code’s Javadoc is included. [file 1471-2105-15-150-S2.zip › additional file 2/ml2db/utils/doc/uk/ac/standrews/utils/main/collectionutils/VectorUtils.html]

VectorUtils


JavaScript is disabled on your browser.


- Overview
- Package
- Class
- Use
- Tree
- Deprecated
- Index
- Help

- Prev Class
- Next Class

- Frames
- No Frames

- All Classes

- Summary:
- Nested |
- Field |
- Constr |
- Method

- Detail:
- Field |
- Constr |
- Method


uk.ac.standrews.utils.main.collectionutils

## Class VectorUtils

- java.lang.Object
- - uk.ac.standrews.utils.main.collectionutils.VectorUtils

- ---

    

  ```
  public class VectorUtils
  extends java.lang.Object
  ```

- - ### Constructor Summary

    Constructors

    Constructor and Description

    `VectorUtils()`
  - ### Method Summary

    Methods

    | Modifier and Type | Method and Description |
    | --- | --- |
    | `static java.util.ArrayList<java.lang.String>` | `listFromVector(java.util.Vector<java.lang.String> vector)` |
    | `static java.util.Vector<java.lang.String>` | `trimList(java.util.Vector<java.lang.String> list)` trim all trailing white spaces from strings in a list (beware: null elements become empty strings) |

    - ### Methods inherited from class java.lang.Object

      `equals, getClass, hashCode, notify, notifyAll, toString, wait, wait, wait`

- - ### Constructor Detail


    - #### VectorUtils

      ```
      public VectorUtils()
      ```
  - ### Method Detail


    - #### listFromVector

      ```
      public static java.util.ArrayList<java.lang.String> listFromVector(java.util.Vector<java.lang.String> vector)
      ```


    - #### trimList

      ```
      public static java.util.Vector<java.lang.String> trimList(java.util.Vector<java.lang.String> list)
      ```

      trim all trailing white spaces from strings in a list (beware: null
      elements become empty strings)

      Parameters:
      :   `list` -

      Returns:
      :   a list of trimmed strings


- Overview
- Package
- Class
- Use
- Tree
- Deprecated
- Index
- Help

- Prev Class
- Next Class

- Frames
- No Frames

- All Classes

- Summary:
- Nested |
- Field |
- Constr |
- Method

- Detail:
- Field |
- Constr |
- Method
